# Identification of the promising accessions of spistan (*Cordia myxa* Roxb.) using morphological and fruit‐related traits

**DOI:** 10.1002/fsn3.3008

**Published:** 2022-07-27

**Authors:** Farhad Mirheidari, Ali Khadivi, Younes Moradi, Simin Paryan

**Affiliations:** ^1^ Department of Horticultural Sciences, Faculty of Agriculture and Natural Resources Arak University Arak Iran

**Keywords:** breeding, *Cordia myxa*, cultivation, fruit, superior genotype

## Abstract

Spistan (*Cordia myxa* Roxb.) is a potentially underutilized fruit plant in arid and semi‐arid regions. It has long been associated with health and nutrition. Morphological diversity of 75 accessions of this species was evaluated. The accessions studied showed significant differences in terms of the characters measured. Ripening date varied from late May to mid‐July. Fruit color was yellow‐cream in 54, light orange in 8, and orange in 13 accessions. The range of fruit weight was 0.71–11.83 g with an average of 3.12, while fruit flesh thickness ranged from 0.63 to 7.86 mm with an average of 2.55, and fruit jelly part thickness varied between 1.34 and 6.40 mm with an average of 2.75. Principal component analysis could describe the evaluated traits as the nine main components that were able to justify 79.04% of total variance. Hierarchical clustering showed that the accessions were placed into two main clusters using the measured traits data, exhibiting a wide range of variability. Based on the traits related to selection of the ideal genotype, such as big fruit size, high fruit flesh thickness with high yield, and longer harvesting period, 11 accessions, including Jangal‐4, Jangal‐9, Rask‐7, Jangal‐5, Jangal‐6, Jangal‐11, Rask‐1, Jangal‐1, Rask‐4, Rask‐5, and Rask‐2, were superior. It is recommended to use the best accessions selected in breeding programs. The commercial orchards of those best accessions should be extensively constructed to take advantage of the high yield of *C. myxa* as a crop and its medicinal properties.

## Graphical abstract

Based on the traits related to select ideal genotype, such as big fruit size, high fruit flesh thickness with high yielding and longer harvesting period, 11 accessions, including Jangal‐4, Jangal‐9, Rask‐7, Jangal‐5, Jangal‐6, Jangal‐11, Rask‐1, Jangal‐1, Rask‐4, Rask‐5, and Rask‐2, were superior. It is recommended to use the best accessions selected in breeding programs. The commercial orchard of those best accessions should be extensively constructed to take advantage of the high yield of C. myxa as a crop and its medicinal properties.

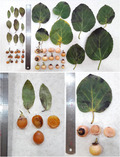

## 1 INTRODUCTION

The family Boraginaceae includes about 2740 species that are in 148 genera. One of the most important genera of this family is *Cordia*, which is in the form of trees and shrubs. There are reported to be 300 species in the genus *Cordia* (Yadav and Yadav, 2013). This scientific name is given to this genus out of respect for the botanist Valerius Cordus (Hussain and Kakoti, 2013). Various parts, such as fruits, leaves, stem bark, seeds, and roots, in the plants of most species of this genus have been used in traditional medicine (Yadav and Yadav, 2013). One of the species in this genus is *C. myxa* Roxb. (Syn. *C. obliqua*, *C. crenata*). Common names of this species include Assyrian plum, lasura, laveda, pidar, panugeri, naruvilli, geduri, spistan, burgund dulu wanan, and ntege. It is in the form of a medium‐sized deciduous tree and its height reaches 10.50 m (Jackson, 1977). Flowering time of this plant begins in the last week of April and continues until late May. Its ripening time is from early July to late August (Gupta and Das Gupta, 2015).

*Cordia myxa* is a fruit tree with high medicinal and nutritional value that grows well in arid and semi‐arid regions. Because it is a multipurpose plant, its value in nutrition and health is of interest (Chandra and Pareek, 1992). Its fresh unripe fruits have a very unpleasant odor and are used for vegetables and pickles when conventional vegetables are not available. This plant plays an important role in the rural economy of arid regions because it can provide food (pickles and vegetables) and fuel wood (Chandra et al., 1994). Its fruits have a high medicinal value and are used for diseases of the chest and urinary passage. Its fruit flesh is used as birdlime, and its kernel is used to cure ringworm (McCann, 1985). Its fruits are rich in carbohydrates and phosphorus and contain 40 mg/100 g of ascorbic acid (Pareek and Sharma, 1993).

The first step in any breeding program is to evaluate and be aware of the variation available. The breeding programs are dependent on the diversity in the nature of the plant in different climatic conditions. Vavilov (1951) was the first to state that high diversity in each plant increases the chances of better selection for the desired traits. Most of the successful tree breeding programs are those that use the right provenance (Zobel and Talbert, 1984). Genetic diversity within and between plant populations can be significantly revealed through morphological assessments. There are very few studies on phenotypic diversity of *C. myxa* in the world (Sivalingam et al., 2012; Meghwal et al., 2014), while there is no information about diversity of this species in Iran. Therefore, the aim of the present study was to determine the phenotypic diversity of this species using the important morphological traits and to select the best plant materials to be used in future breeding or cultivation programs.

## 2 MATERIAL AND METHODS

### 2.1 Plant material

Morphological diversity of 75 accessions of *C. myxa* was evaluated from three areas of Sistan‐va‐Baluchestan and one area of Khuzestan provinces, Iran. Geographical coordinates and altitude corresponding to collection sites are shown in Table 1. The appropriate distances were considered between the accessions in each collection site to avoid the possibility of sampling and collecting clones of the selected trees.

**TABLE 1:** Geographical description for collection sites of *C. myxa* accessions studied

No.	Province	Area	Latitude (N)	Longitude (E)	Altitude (m)	Sample size
1	Sistan‐va‐Baluchestan	Rask	26°13′08″	61°24′38″	378	9
2	Sistan‐va‐Baluchestan	Jangal	26°17′27″	60°43′23″	453	11
3	Khuzestan	Izeh	31°49′59″	49°52′16″	836	34
4	Sistan‐va‐Baluchestan	Ghasrghand	26°14′09″	60°44′25″	528	21

### 2.2 The characteristics evaluated

A total of 48 quantitative and qualitative morphological and pomological traits (Table 2) were used for phenotypic evaluations. The traits related to dimensions of different organs were measured using a digital caliper. Bunch weight, fruit weight, and fruit stone weight were measured using an electronic balance with 0.01 g precision. Also, the remaining characteristics were qualitatively estimated based on rating and coding (Table 3).

**TABLE 2:** Statistical descriptive parameters for morphological traits used to study *C. myxa* accessions

No.	Trait	Abbreviation	Unit	Min	Max	Mean	SD	CV (%)
1	Tree growth habit	TGH	Code	1	9	4.39	2.67	60.84
2	Tree growth vigor	TGV	Code	1	5	3.77	1.35	35.84
3	Tree height	TH	Code	1	5	3.83	1.32	34.44
4	Branching	Br	Code	1	5	3.91	1.24	31.79
5	Branch density	BrD	Code	1	5	4.28	1.07	25.07
6	Branch flexibility	BrF	Code	1	5	3.13	1.62	51.82
7	Trunk diameter	TrDi	Code	1	5	3.51	1.44	40.94
8	Trunk color	TrC	Code	1	7	2.44	1.70	69.47
9	Canopy density	CaDe	Code	1	5	3.00	1.68	55.90
10	Tendency to form suckers	TeS	Code	0	1	0.05	0.35	700.00
11	Leaf density	LDe	Code	1	5	4.25	1.13	26.54
12	Leaf length	LLe	mm	45.28	135.36	77.67	20.47	26.36
13	Leaf width	LWi	mm	23.12	113.32	58.99	22.90	38.81
14	Leaf thickness	LTh	mm	0.16	1.35	0.54	0.24	44.11
15	Petiole length	PeLe	mm	11.10	49.33	27.60	8.78	31.81
16	Petiole width	PeWi	mm	0.88	3.97	2.25	0.90	39.81
17	Leaf apex shape	LAp	Code	1	5	3.13	1.59	50.73
18	Leaf base shape	LBa	Code	1	7	3.48	1.91	54.97
19	Leaf shape	LSh	Code	1	7	4.33	1.98	45.77
20	Leaf margin	LMa	Code	0	1	0.69	0.46	67.25
21	Leaf serration shape	LSeSh	Code	1	7	3.56	2.22	62.33
22	Leaf serration depth	LSeDep	Code	0	5	1.68	1.61	95.95
23	Leaf upper surface color	LUSuC	Code	3	5	4.41	0.92	20.79
24	Leaf lower surface color	LLoSuC	Code	1	3	2.17	0.99	45.67
25	Ripening date	RiDa	Date	Late May	Mid‐July	3.85	2.26	58.65
26	Fruit density	FrD	Code	1	5	3.93	1.41	35.83
27	Bunch weight	BuWe	g	4.69	78.63	17.12	14.45	84.43
28	Bunchlet no. per bunch	BultNoBu	Number	2	7	4.21	0.93	22.19
29	Fruit no. per bunch	FrNoBu	Number	3	15	6.84	2.65	38.68
30	Fruit shape	FrSh	Code	1	7	4.47	2.01	44.94
31	Fruit length	FrLe	mm	12.36	24.68	16.76	3.65	21.76
32	Fruit diameter	FrDi	mm	9.63	29.36	15.46	5.65	36.56
33	Fruit stalk length	FrStLe	mm	2.28	8.43	4.05	1.42	35.01
34	Fruit stalk diameter	FrStDi	mm	1.10	3.81	2.49	0.71	28.76
35	Calyx diameter	CaDi	mm	3.55	18.75	9.21	4.45	48.36
36	Calyx color	CaCo	Code	1	9	3.61	2.50	69.34
37	Calyx margin	CaMa	Code	1	5	2.68	1.19	44.29
38	Calyx shape	CaSh	Code	1	5	3.24	1.35	41.79
39	Fruit weight	FrWe	g	0.71	11.83	3.12	3.27	104.73
40	Fruit color	FrCo	Code	1	5	1.91	1.55	81.26
41	Fruit taste	FrTa	Code	1	3	2.36	0.94	39.79
42	Fruit flesh firmness	FrFlFi	Code	1	5	2.25	1.35	59.87
43	Fruit flesh thickness	FrFlTh	mm	0.63	7.86	2.55	1.94	76.36
44	Fruit jelly part thickness	FrJPTh	mm	1.34	6.40	2.75	0.94	34.22
45	Fruit stone length	FrSnLe	mm	8.64	15.12	10.84	1.56	14.40
46	Fruit stone width	FrSnWi	mm	5.39	16.44	9.05	2.38	26.32
47	Fruit stone thickness	FrSnTh	mm	4.06	8.63	6.16	0.86	13.88
48	Fruit stone weight	FrSnWe	g	0.11	1.20	0.35	0.25	69.84

**TABLE 3:** Frequency distribution for the measured qualitative morphological characters in the studied *C. myxa* accessions

	Frequency (no. of accessions)					
Character	0	1	3	5	7	9
Tree growth habit	—	Weeping (16)	Spreading (26)	Open (5)	Semi‐erect (21)	Erect (7)
Tree growth vigor	—	Low (8)	Moderate (30)	High (37)	—	—
Tree height	—	Low (7)	Moderate (30)	High (38)	—	—
Branching	—	Low (5)	Moderate (31)	High (39)	—	—
Branch density	—	Low (2)	Moderate (23)	High (50)	—	—
Branch flexibility	—	Low (22)	Moderate (26)	High (27)	—	—
Trunk diameter	—	Low (12)	Moderate (32)	High (31)	—	—
Trunk color	—	Light brown (38)	Brown‐gray (22)	Brown (13)	Dark brown (2)	—
Canopy density	—	Low (26)	Moderate (23)	High (26)	—	—
Tendency to form suckers	Absent (71)	Present (3)	—	—	—	—
Leaf density	—	Low (3)	Moderate (22)	High (50)	—	—
Leaf apex shape	—	Acute (21)	Obtusely acuminate (28)	Obtuse (26)	–	–
Leaf base shape	—	Acute (14)	Rounded (42)	Truncate (6)	Cordate (13)	—
Leaf shape	—	Lanceolate (15)	Oblong (8)	Ovate (39)	Cordate (13)	—
Leaf margin	Absent (23)	Present (52)	—	–	—	—
Leaf serration shape	—	Entire (23)	Undulate (23)	Dentate (14)	Serrate (15)	—
Leaf serration depth	None (23)	Low (22)	Moderate (23)	High (7)	—	—
Leaf upper surface color	—	—	Green (22)	Dark green (53)	—	—
Leaf lower surface color	—	Light green (31)	Green (44)	—	—	—
Ripening date	—	Late May (20)	Early June (21)	Early July (16)	Mid‐July (18)	—
Fruit density	—	Low (9)	Moderate (22)	High (44)	—	—
Fruit shape	—	Oblate (12)	Round (14)	Oblong (31)	Obovate (18)	—
Calyx color	—	Light green (27)	Green‐cream (15)	Cream (22)	Brown (5)	Dark brown (6)
Calyx margin	—	Narrow‐dentate (20)	Dentate (47)	Broad‐dentate (8)	—	—
Calyx shape	—	Broad‐bowl (13)	Bowl (40)	Bell (22)	—	—
Fruit color	—	Yellow‐cream (54)	Light orange (8)	Orange (13)	—	—
Fruit taste	—	Astringent‐sweet (24)	Sweet (51)	—	—	—
Fruit flesh firmness	—	Low (36)	Moderate (31)	High (8)	—	—

### 2.3 Statistical analysis

Analysis of variance (ANOVA) was performed to evaluate the variation among the accessions based on the traits measured using SAS software (SAS Institute, 1990). Simple correlations between traits were determined using Pearson correlation coefficients (SPSS Inc., Norusis, 1998). Principal component analysis (PCA) was used to investigate the relationship among the accessions and determine the main traits effective in accession segregation using SPSS software. Hierarchical cluster analysis (HCA) was performed using Ward's method and Euclidean coefficient using PAST software (Hammer et al., 2001). The first and second principal components (PC1/PC2) were used to create a scatter plot with PAST software.

## 3 RESULTS AND DISCUSSION

The accessions studied showed significant differences in terms of the characteristics measured. The least coefficient of variance (CV) belonged to fruit stone thickness (13.88%) and then fruit stone length (14.40%). The CV of 46 out of 48 characters recorded was more than 20.00%: tendency to form suckers exhibited the highest CV (700%) followed by fruit weight (104.73%), leaf serration depth (95.95%), bunch weight (84.43%), fruit color (81.26%), and fruit flesh thickness (76.36%) (Table 2). For comparison, Meghwal et al. (2014) reported 45.03% as CV of bunch weight and 30.34% as CV of fruit weight in a *C. myxa* germplasm from India.

Tree growth habit was highly variable and included weeping (16 accessions), spreading (26), open (5), semi‐erect (21), and erect (7). Tree growth vigor, tree height, branching, branch density, branch flexibility, and leaf density were dominantly high (Table 3). The majority of accessions did not show the tendency to form suckers (71). Leaf shape showed high diversity, including lanceolate (15 accessions), oblong (8), ovate (39), and cordate (13). For comparison, Sivalingam et al. (2012) reported oval, oblong, obtuse, and cordate shapes for leaf of a *C. myxa* germplasm from India. Also, leaf serration shape was highly variable, including entire (23 accessions), undulate (23), dentate (14), and serrate (15) (Table 3). The range of leaf‐related traits was as follows: leaf length: 45.28–135.36 mm, leaf width: 23.12–113.32 mm, leaf thickness: 0.16–1.35 mm, petiole length: 11.10–49.33 mm, and petiole width: 0.88–3.97 mm (Table 2). For comparison, Sivalingam et al. (2012) reported the range of 52.00–125.30 mm for leaf length and 36.70–120.00 mm for leaf width in a *C. myxa* germplasm from India. Nagar and Fageria (2006) reported that leaf size was sufficient for indirect selection of genotypes as it had positive correlation with many of the horticulturally useful traits.

Ripening date varied from late May to mid‐July. Fruit density was low in 9, moderate in 22, and high in 44 accessions. The range of bunch weight, bunchlet number per bunch, and fruit number per bunch was as follows: 4.69–78.63 g, 2–7, and 3–15, respectively (Table 2). Meghwal et al. (2014) reported the range of 14.10–137.40 g for bunch weight and 4.62–14.80 for fruit number per bunch in a *C. myxa* germplasm from India. Four types of fruit shape were observed, including oblate (12 accessions), round (14), oblong (31), and obovate (18). Sivalingam et al. (2012) reported oblong and round shapes for fruit of a *C. myxa* germplasm from India.

Strong variability was observed in terms of calyx color, including light green (27 accessions), green‐cream (15), cream (22), brown (5), and dark brown (6). Fruit color was yellow‐cream in 54, light orange in 8, and orange in 13 accessions. Fruit taste was astringent‐sweet in 24 and sweet 51 accessions (Table 3). Fruit length ranged from 12.36 to 24.68 mm, fruit diameter varied between 9.63 and 29.36 mm, fruit stalk length ranged from 2.28 to 8.43 mm, and fruit stalk diameter varied between 1.10 and 3.81 mm (Table 2). Sivalingam et al. (2012) reported the range of 13.90–25.00 mm for fruit length and 8.70–25.00 mm for fruit diameter in a *C. myxa* germplasm from India.

The range of fruit weight was 0.71–11.83 g with an average of 3.12, while fruit flesh thickness ranged from 0.63 to 7.86 mm with an average of 2.55, and fruit jelly part thickness varied between 1.34 and 6.40 mm with an average of 2.75 (Table 2). Sivalingam et al. (2012) reported the range of 0.68–8.11 g for fruit weight in a *C. myxa* germplasm from India, while Meghwal et al. (2014) reported the range of 1.96–10.50 g for fruit weight in a *C. myxa* germplasm from India.

**FIGURE 1:**
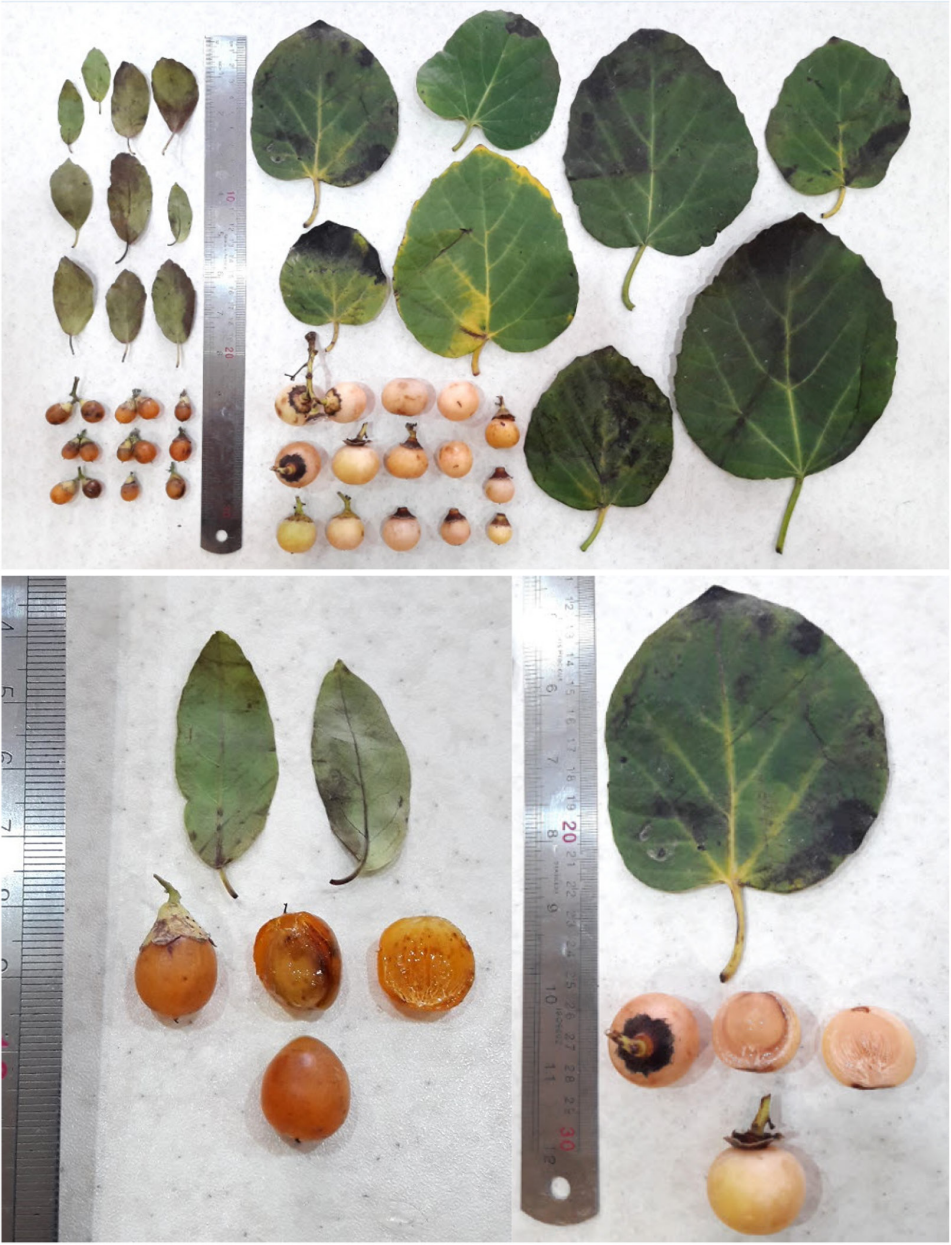
The pictures of leaves and fruits of *C. myxa* accessions studied

The range of fruit stone‐related traits was as follows: stone length: 8.64–15.12 mm, stone width: 5.39–16.44 mm, stone thickness: 4.06–8.63 mm, and stone weight: 0.11–1.20 g. Sivalingam et al. (2012) reported the range of 8.70–14.60 mm for stone length, 7.20–13.30 mm for stone width, 5.33–7.80 mm for stone thickness, and 0.11–0.44 g for stone weight in a *C. myxa* germplasm from India. The pictures of leaves and fruits of the studied *C. myxa* accessions are shown in Figure 1.

PCA could describe the evaluated traits as the nine main components that were able to justify 79.04% of total variance (Table 4). The PC1 was correlated with 19 characters, including leaf length, leaf width, leaf thickness, petiole width, leaf base shape, leaf shape, bunch weight, fruit shape, fruit length, fruit diameter, fruit stalk diameter, calyx shape, fruit weight, fruit color, fruit flesh thickness, fruit jelly part thickness, fruit stone length, fruit stone width, and fruit stone weight, explaining for 31.12% of total variance. In PC2, 12 traits, including canopy density, petiole length, leaf apex shape, leaf margin, leaf serration shape, leaf upper surface color, leaf lower surface color, ripening date, calyx diameter, calyx color, fruit taste, and fruit stone thickness, were found, accounting for 19.58% of total variance. The PC1 and PC2 played a major role in distinguishing the accessions studied. Three characters, including tree growth vigor, tree height, and trunk diameter, were placed in the PC3 and accounted for 6.42% of total variance. Each of the remaining PCs (PC4‐PC9) displayed low variance (<5.00%) and thus had minor roles in distinguishing the accessions.

**TABLE 4:** Eigenvalues of the principal component axes from the PCA of the morphological characters in the studied *C. myxa* accessions

Trait	Component								
	1	2	3	4	5	6	7	8	9
Tree growth habit	0.19	0.06	−0.20	0.10	0.73**	0.20	−0.06	0.19	0.02
Tree growth vigor	0.26	−0.25	0.66**	−0.17	−0.01	0.18	0.02	−0.04	−0.19
Tree height	−0.03	0.07	0.87**	−0.11	−0.04	0.07	0.03	0.02	0.07
Branching	0.24	−0.32	0.44	−0.03	0.11	−0.35	0.32	0.02	0.00
Branch density	0.06	−0.43	0.44	0.29	−0.18	−0.21	0.23	−0.17	−0.21
Branch flexibility	0.09	−0.32	−0.13	0.03	−0.69**	0.01	−0.10	0.25	0.17
Trunk diameter	0.01	−0.14	0.70**	−0.08	0.01	−0.25	−0.23	−0.14	0.22
Trunk color	−0.01	0.11	0.13	0.19	−0.01	0.81**	0.11	0.13	−0.03
Canopy density	−0.01	0.70**	0.17	0.10	−0.09	−0.39	0.18	0.05	−0.13
Tendency to form suckers	−0.01	−0.11	0.04	0.03	0.02	−0.02	−0.85**	−0.03	0.00
Leaf density	−0.08	0.03	0.27	0.15	−0.29	−0.65**	0.12	0.07	−0.15
Leaf length	0.78**	−0.01	0.04	0.27	0.19	−0.02	−0.17	0.24	−0.12
Leaf width	0.89**	−0.15	0.12	0.09	0.09	0.03	−0.19	0.21	−0.08
Leaf thickness	0.71**	−0.05	0.18	−0.11	0.21	0.09	0.08	0.25	−0.24
Petiole length	0.48	−0.64**	0.16	−0.01	−0.08	−0.11	−0.36	0.09	−0.12
Petiole width	0.86**	0.14	0.17	0.00	0.13	0.01	−0.07	0.30	−0.04
Leaf apex shape	0.39	−0.61**	0.22	−0.49	−0.08	−0.13	−0.03	0.09	0.04
Leaf base shape	0.84**	0.06	0.03	−0.13	0.08	0.06	−0.03	−0.11	0.08
Leaf shape	0.85**	−0.32	0.11	−0.15	0.04	0.10	−0.08	−0.04	0.02
Leaf margin	0.11	0.66**	−0.17	0.59	0.13	0.12	−0.08	−0.03	−0.04
Leaf serration shape	−0.26	0.61**	−0.14	0.60	−0.04	−0.03	0.00	−0.01	−0.04
Leaf serration depth	−0.09	0.31	−0.14	0.83**	0.04	0.05	−0.03	−0.02	0.17
Leaf upper surface color	0.04	0.81**	0.01	−0.03	−0.04	0.16	−0.17	0.17	−0.09
Leaf lower surface color	0.09	0.87**	−0.05	0.24	0.13	0.02	0.12	0.13	−0.01
Ripening date	−0.10	−0.90**	0.09	−0.17	−0.04	−0.01	−0.03	−0.08	0.01
Fruit density	−0.14	−0.11	0.02	0.08	−0.16	0.08	−0.05	0.07	0.63**
Bunch weight	0.84**	0.28	−0.14	0.03	−0.04	0.09	0.07	−0.03	0.23
Bunchlet no. per bunch	0.28	0.21	−0.24	−0.07	0.00	0.12	0.06	0.72**	0.22
Fruit no. per bunch	−0.37	−0.30	−0.47	0.02	−0.03	0.21	−0.01	0.07	0.47
Fruit shape	−0.88**	0.00	−0.10	0.08	−0.02	0.09	−0.02	−0.22	0.10
Fruit length	0.87**	0.41	0.00	0.02	0.08	0.01	0.07	−0.02	−0.12
Fruit diameter	0.91**	0.38	0.01	0.01	0.08	−0.03	0.08	0.05	−0.06
Fruit stalk length	0.56	0.21	0.16	0.14	0.41	−0.01	−0.14	0.20	−0.14
Fruit stalk diameter	0.86**	−0.06	−0.01	−0.20	−0.11	−0.09	0.10	0.07	−0.08
Calyx diameter	0.53	0.78**	−0.02	0.12	0.20	0.03	0.13	0.04	−0.01
Calyx color	0.29	0.78	0.07	−0.02	0.08	−0.05	0.11	0.01	0.01
Calyx margin	−0.03	0.30	0.27	0.00	0.26	−0.16	0.36	0.06	0.55
Calyx shape	−0.85**	0.18	−0.09	0.15	0.02	0.12	0.05	−0.05	0.08
Fruit weight	0.88**	0.43	−0.01	0.03	0.09	0.00	0.10	0.04	−0.03
Fruit color	−0.68**	0.56	−0.16	0.16	0.09	−0.01	0.08	−0.07	0.13
Fruit taste	0.07	0.77**	−0.11	0.05	0.21	0.09	−0.10	−0.10	0.01
Fruit flesh firmness	0.48	0.59	0.02	0.10	−0.02	−0.02	0.08	0.21	−0.07
Fruit flesh thickness	0.63**	0.45	0.09	−0.01	0.31	−0.07	0.08	0.34	−0.25
Fruit jelly part thickness	0.67**	−0.41	0.24	−0.14	−0.09	−0.17	−0.02	−0.22	−0.02
Fruit stone length	0.86**	0.15	−0.03	0.06	−0.02	0.10	0.07	−0.13	−0.05
Fruit stone width	0.93**	0.16	−0.10	−0.03	−0.08	−0.03	0.04	−0.13	−0.03
Fruit stone thickness	0.42	0.64**	−0.25	0.06	−0.04	0.02	0.15	−0.28	0.09
Fruit stone weight	0.81**	0.39	−0.12	0.04	−0.13	0.06	0.10	−0.24	0.11
Total	14.94	9.40	3.08	2.27	1.83	1.82	1.55	1.53	1.53
% of Variance	31.12	19.58	6.42	4.72	3.82	3.79	3.23	3.18	3.18
Cumulative %	31.12	50.70	57.12	61.84	65.66	69.45	72.67	75.86	79.04

** Eigenvalues ≥0.61 are significant at the *p* ≤ 0.01 level.

**FIGURE 2:**
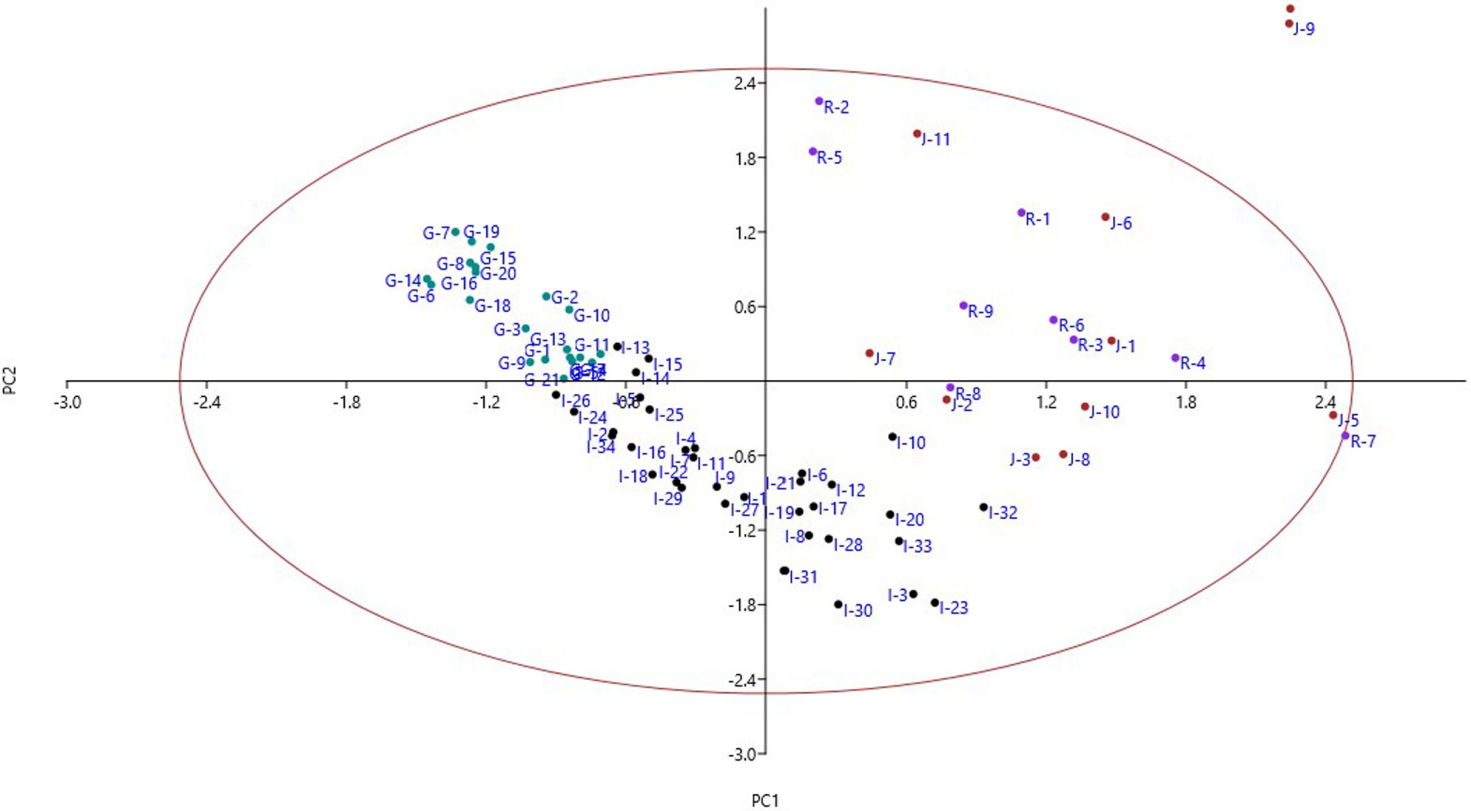
Scatter plot for the studied *C. myxa* accessions based on PC1/PC2. The symbols represent the accessions of each area in the plot, including Jangal (J), Rask (R), Izeh (I), and Ghasrghand (G)

**FIGURE 3:**
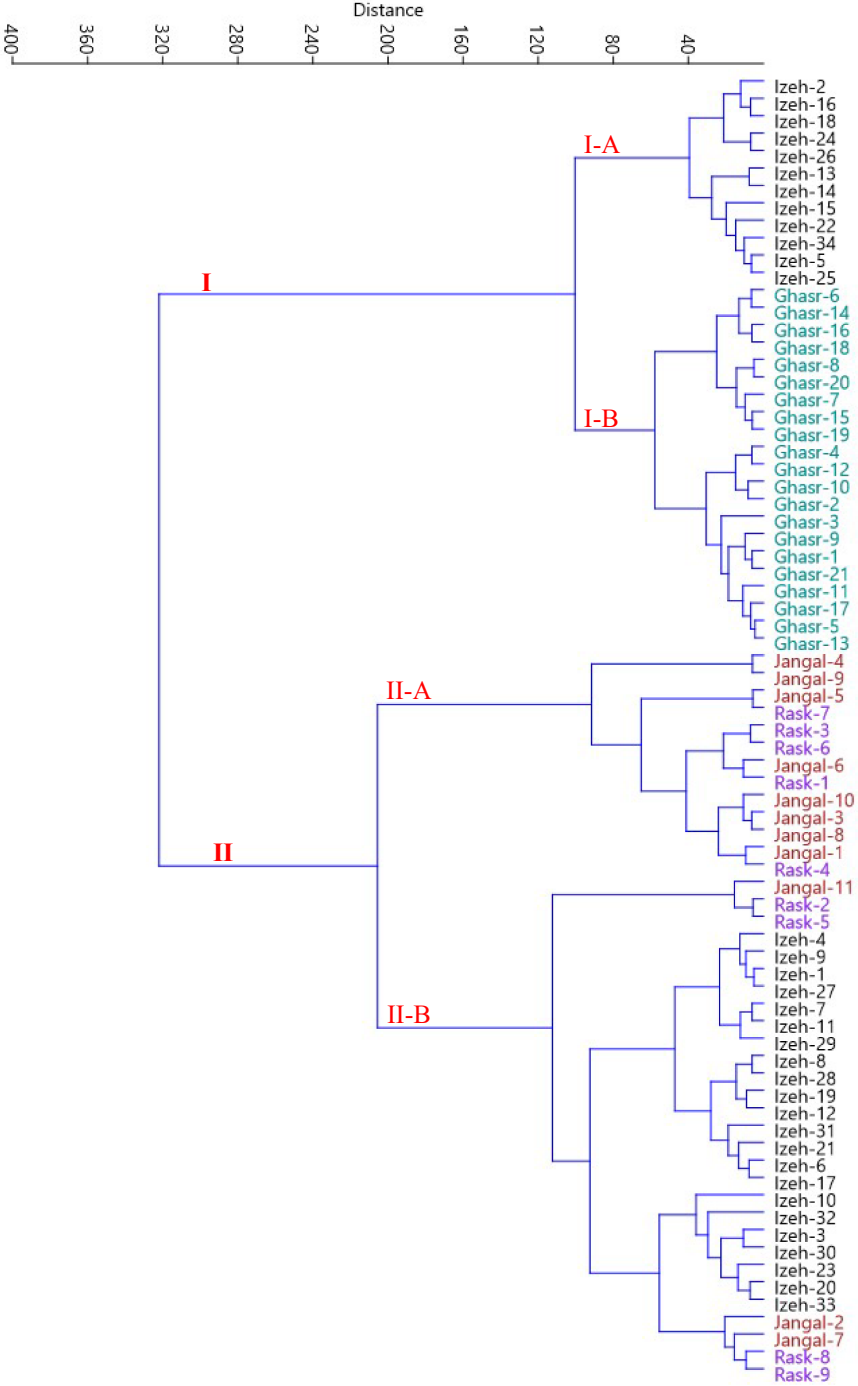
Ward cluster analysis of the studied *C. myxa* accessions based on morphological traits using Euclidean distances

**FIGURE 4:**
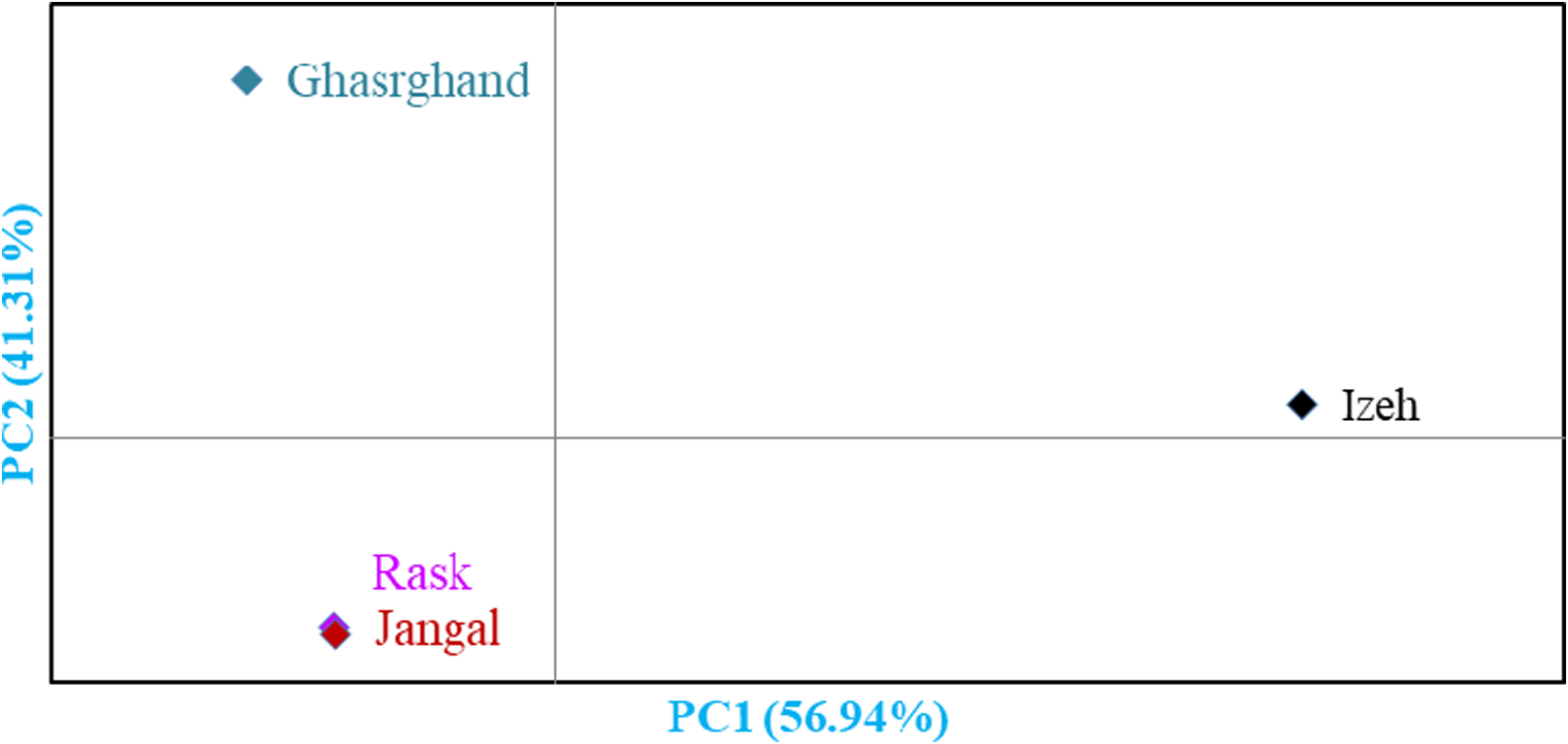
Bi‐plot for the studied populations of *C. myxa* based on the morphological characters

Scatter plot analysis was performed using PC1 and PC2, which accounted for 50.70% of total variance (Figure 2). The accessions that were in close proximity were more similar in terms of effective traits in PC1 and PC2 and were placed in the same group. Also, hierarchical clustering was performed based on the dissimilarity of the accessions. The accessions were clustered into two main clusters using the measured traits data (Figure 3). The first cluster contained 33 accessions which formed two subclusters. Subcluster I‐A included 12 accessions of Ized area, while subcluster I‐B consisted of all 21 accessions of Ghasrghand area. The second clusters (II) consisted of the rest of accessions, which formed two subclusters. Subcluster II‐A included eight accessions of Jangal and five accessions of Rask areas, while subcluster II‐B consisted of 3 accessions of Jangal, 4 accessions of Rask, and 22 accessions of Ized areas. Besides, according to the population analysis (Figure 4), the studied areas were placed into three groups. The Ghasrghand population was placed in the first group, while the Ized population formed the second group. Also, the Rask and Jangal populations were placed in the third group.

The studied accessions exhibited a wide range of variability for most of the characteristics recorded. Variation refers to the observable differences among individuals for a particular trait. These differences may be partly due to genotypic factors and partly due to environmental effects. The combined reflections of both factors are the phenotypic effect. For the proper utilization of observed variation in a species, it is prerequisite to know the extent of variation and also whether it is due to the genetic or the environmental factors. Hence, information on variability among the desirable characteristics and their correlation is vital for any breeding program (Johnson et al., 1955). Therefore, a species exhibiting a wide range of variability (in terms of a characteristic's value and high standard deviation) offers ample scope for undertaking screening for the desired traits (Nagar et al., 2013).

The observed variation in a characteristic is partly composed of genetic (heritable) variation and partly of nonheritable. The proportion of total variation, which is due to genetic differences, is termed as heritability in a broad sense (Nagar et al., 2013). Heritability provides a measure of genetic variation upon which all the possibilities of changing the genetic composition of the species depend. Genetic advance refers to the improvement in the mean genotypic values of the selected genotypes over the populations. The genetic coefficient of variation indicates the range and magnitude of genetic variability existing between the characteristics, whereas the observed variation in a group of individuals is known as phenotypic coefficient of variation. In the present investigation, the result obtained for the different characteristics with regard to the variability parameters indicates that values have a wide range depicting the presence of high amount of variation. Those characteristics having high heritability coupled with high genetic advance as a percentage of mean indicate that the improvement in these traits can be made through direct selection (Nagar et al., 2013). The *C. myxa* has high potential to be exploited at the industrial or commercial level (Chandra and Pareek 1992) which requires an ideal genotype having big fruit size (9.00–12.00 g), high fruit flesh thickness with high yield, and a long harvesting period (Samadia 2005).

## 4 CONCLUSION

Since there are no known commercial cultivars in *C. myxa*, improvement work for developing new cultivars should be undertaken. Based on the traits related to the selection of the ideal genotype, such as big fruit size (9.00–12.00 g), high fruit flesh thickness with high yield, and longer harvesting period, 11 accessions, including Jangal‐4, Jangal‐9, Rask‐7, Jangal‐5, Jangal‐6, Jangal‐11, Rask‐1, Jangal‐1, Rask‐4, Rask‐5, and Rask‐2, were superior. It is recommended to use the best accessions selected in breeding programs. The commercial orchards of those best accessions should be extensively constructed to take advantage of the high yield of *C. myxa* as a crop.

## Funding information

None.

## CONFLICT OF INTEREST

The authors declare no conflict of interest.

## RESEARCH INVOLVING HUMAN PARTICIPANTS AND/OR ANIMALS

None.

## INFORMED CONSENT

None.

## Data Availability

The data that support the findings of this study are available from the corresponding author upon reasonable request.

## References

[fsn33008-bib-0001] Chandra, A. , Chandra, A. , & Gupta, I. C. (1994). Cordia myxa. In Arid fruit research (p. 302). Scientific Publisher.

[fsn33008-bib-0002] Chandra, A. , & Pareek, C. S. (1992). Lasoda (*Cordia myxa* L.)—A potential fruit crop in Jaisalmer district of western Rajasthan. Agricultural Science Digest, 12(1), 11–12.

[fsn33008-bib-0003] Gupta, R. , & Das Gupta, G. (2015). A review on plant Cordiaobliqua Willd. (clammy cherry). Pharmacognosy Reviews, 9(18), 127–131. 26392710 10.4103/0973-7847.162124 PMC4557235

[fsn33008-bib-0004] Hammer, Ø. , Harper, D. A. T. , & Ryan, P. D. (2001). PAST: Paleontological statistics software package for education and data analysis. Palaeontologia Electronica, 4(1), 9. http://palaeoelectronica.org/2001_1/past/issue1_01.htm

[fsn33008-bib-0005] Hussain, N. , & Kakoti, B. B. (2013). Review on ethnobotany and phytopharmacology of *Cordia dichotoma* . Journal of Drug Delivery & Therapeutics, 3(1), 110–113.

[fsn33008-bib-0006] Jackson, B. D. (1977). Index kewensis (Vol. 1, p. 614). Calrendon Press.

[fsn33008-bib-0007] Johnson, H. W. , Robinson, H. F. , & Comstock, R. E. (1955). Estimates of genetic and environmental variability in soybean. Agronomy Journal, 47, 314–318.

[fsn33008-bib-0008] McCann, C. (1985). Trees of India: A popular handbook (p. 23). Periodical Expert Book Agency.

[fsn33008-bib-0009] Meghwal, P. R. , Singh, A. , Kumar, P. , & Morwal, B. R. (2014). Diversity, distribution and horticultural potential of *Cordia myxa* L.: A promising underutilized fruit species of arid and semi arid regions of India. Genetic Resources and Crop Evolution, 61, 1633–1643.

[fsn33008-bib-0010] Nagar, B. L. , Fageria, M. S. , & Pareek, S. (2013). Genetic variation for physicochemical characteristics in Lehsua (*Cordia myxa* L.). African Journal of Agricultural Research, 8, 5047–5050.

[fsn33008-bib-0011] Nagar, B. L. , & Fageria, M. S. (2006). Genetic divergence in Lehsua (*Cordia myxa* Roxb.). Indian Journal of Genetics and Plant breeding, 66, 67–68.

[fsn33008-bib-0012] Norusis, M. J. (1998). SPSS/PC advanced statistics. SPSS Inc.

[fsn33008-bib-0013] Pareek, O. P. , & Sharma, S. (1993). Underutilized fruits. Ind. Hort., 38, 47–56.

[fsn33008-bib-0014] Samadia, D. K. (2005). Genetic variability studies in Lasora (*Cordia myxa* Roxb.). Indian Journal of Plant Genetic Resources, 18, 236–240.

[fsn33008-bib-0015] SAS® Procedures . (1990). Version 6 (3rd ed.). SAS Institute.

[fsn33008-bib-0016] Sivalingam, P. N. , Singh, D. , & Chauhan, S. (2012). Morphological and molecular diversity of an underutilized fruit crop *Cordia myxa* L. germplasm from arid region of Rajasthan. Genetic Resources and Crop Evolution, 59, 305–316.

[fsn33008-bib-0017] Vavilov, N. I. (1951). The origin, variation, immunity and breeding of cultivated plants. (translated from Russian by Chester, KS). Chronica Bot., 13, 1–364.

[fsn33008-bib-0018] Yadav, R. , & Sk, Y. (2013). Evaluation of antimicrobial activity of seeds and leaves of Cordia obliqua wild against some oral pathogens. Indo American Journal of Pharmaceutical Research, 3(8), 6035–6043.

[fsn33008-bib-0019] Zobel, B. , & Talbert, J. (1984). Applied forest tree improvement (p. 505). John Wiley and Sons.

